# Impact of Drought on Soluble Sugars and Free Proline Content in Selected *Arabidopsis* Mutants

**DOI:** 10.3390/biology9110367

**Published:** 2020-10-29

**Authors:** Libero Gurrieri, Martina Merico, Paolo Trost, Giuseppe Forlani, Francesca Sparla

**Affiliations:** 1 Department of Pharmacy and Biotechnology FaBiT, University of Bologna, 40126 Bologna, Italy; libero.gurrieri2@unibo.it (L.G.); martina.merico@hotmail.it (M.M.); paolo.trost@unibo.it (P.T.); 2 Department of Life Science and Biotechnology, University of Ferrara, 44121 Ferrara, Italy; flg@unife.it

**Keywords:** drought stress, metabolic adjustment, proline, soluble sugars, water-insoluble carbohydrates

## Abstract

**Simple Summary:**

Drought has severe effects on plants, negatively impacting economic, agricultural and environmental processes. Depending on the duration and the strength of water stress conditions, plants adjust a series of physiological, cellular, and molecular mechanisms aimed at providing a correct stress response and, if possible, at establishing stress tolerance. The model plant *Arabidopsis thaliana* is one of the best tools for analyzing the involvement of specific genes in the response to drought. Thanks to this tool, the role of two genes encoding enzymes involved in sugars metabolism, and one gene encoding an enzyme involved in proline synthesis, have been investigated. In addition to suggesting an interaction between the metabolism of proline and that of soluble sugars, results broaden our understanding of the predominant role played by the accumulation of soluble sugars in counteracting mild osmotic stress.

**Abstract:**

Water shortage is an increasing problem affecting crop yield. Accumulation of compatible osmolytes is a typical plant response to overcome water stress. Sucrose synthase 1 (SUS1), and glucan, water dikinase 2 (GWD2) and δ^1^-pyrroline-5-carboxylate synthetase 1 (P5CS1) are members of small protein families whose role in the response of *Arabidopsis thaliana* plants to mild osmotic stress has been studied in this work. Comparative analysis between wild-type and single loss-of-function T-DNA plants at increasing times following exposure to drought showed no differences in the content of water-insoluble carbohydrate (i.e., transitory starch and cell wall carbohydrates) and in the total amount of amino acids. On the contrary, water-soluble sugars and proline contents were significantly reduced compared to wild-type plants regardless of the metabolic pathway affected by the mutation. The present results contribute to assigning a physiological role to GWD2, the least studied member of the GWD family; strengthening the involvement of SUS1 in the response to osmotic stress; showing a greater contribution of soluble sugars than proline in osmotic adjustment of *Arabidopsis* in response to drought. Finally, an interaction between proline and soluble sugars emerged, albeit its nature remains speculative and further investigations will be required for complete comprehension.

## 1. Introduction

Drought is one of the main factors affecting agricultural yields in both irrigated and rainfed systems [1]. In recent decades, much effort has been made to study plant responses to drought in order to address the present and future risks associated with climate change [2,3]. In plants, drought rapidly causes an osmotic imbalance. Due to low water content, soil water potential decreases counteracting the osmotic potential generated by plants, with consequent reduction in water uptake.

Plants gradually adapt to drought through several processes occurring at morphological, physiological and molecular levels. During the early stage of water deficit, plants reduce water loss by limiting transpiration through stomata closure [4,5]. As stress severity increases, plants face drought through the accumulation of high intracellular levels of osmoprotectant compounds to protect cellular components and to restore the osmotic balance [6], altering their metabolism to better cope with stress rather than growth (for a recent review, see [7]). Among these compatible osmolytes are proline, glycine betaine, polyamines and sugars [7]. At the same time, the oxidative events resulting from continuous exposure to stress lead to the induction of numerous genes that code for antioxidant enzymes, e.g., superoxide dismutase, ascorbate peroxidase, catalase, and glutathione reductase [8,9]. Finally, if drought is not relieved, plants cannot endure further stress because of their sessile lifestyle, and die.

Several enzymes involved in either sugar or amino acid metabolism seem to play a role in the multifaceted response of plants to drought. Sucrose synthases (SUSs) are glycosyl transferase enzymes located both in the cytoplasm and associated with the plasma membrane (for a recent review see [10]). With a marked preference for UDP over ADP [11], SUS catalyzes the reversible cleavage of sucrose to fructose and UDP/ADP-glucose. The forward reaction is favored at slightly acidic pH values (5.5 to 7.5), whereas the reverse reaction prevails at higher pH values [12]. Since sucrose is the main sugar exported from source tissues [13], the SUS family is presumably responsible for the plasticity of sucrose metabolism and in general to play a key role in phloem downloading and sugar metabolism in sink tissues. In *Arabidopsis* 6 genes, the *SUS* gene family is composed [14]. According to [10], these genes are divided into three clades, with *SUS5* and *SUS6* belonging to most ancient clade III, *SUS2* and *SUS3* to clade II, and *SUS1* and *SUS4* to clade I. Both single and double *Arabidopsis* mutants lacking pairs of isoforms of the same clade exhibit phenotypes similar to wild-type plants, suggesting a strong functional redundancy between and within clades [15]. The only exception is a retarded growth and sucrose accumulation observed in response to hypoxia in *sus1*/*sus4* double mutant, suggesting a specialization of clade I genes in response to stress [15]. This hypothesis is further supported by the induction of *SUS1* and *SUS4* orthologous in tobacco plants exposed to drought [16].

Glucan, water dikinases (GWDs) catalyze the phosphorylation of amylopectin chains at the surface of the starch granules. Depending on the substrate, GWDs are distinguished in GWD and phosphoglucan, water dikinase (PWD). The former phosphorylates the C-6 positions of a not phosphorylated amylopectin chain [17] while the latter phosphorylates the C-3 position of a pre-phosphorylated amylopectin chain [18,19]. In dicotyledons, three members form the GWD family [20]. In *Arabidopsis*, GWD1 and PWD are localized in the chloroplast stroma [18,21] and play a central role in transitory starch degradation [18,19,22]. GWD2 is the third member of the family and showed an unexpected cytosolic localization, excluding a role in starch metabolism [23]. The physiological function of GWD2 is still unclear. Under non-stressful conditions, adult *gwd2* plants displayed normal growth, probably due to a restricted expression in companion cells of the phloem at the onset of senescence [23]. A stronger phenotype was instead observed in *gwd2* seeds that appeared shrunken and less prone to germination [24].

In higher plants, proline synthesis occurs mainly in the cytosol from glutamate. A two-step reaction catalyzed by δ^1^-pyrroline-5-carboxylate synthetase (P5CS) reduces glutamate to glutamate semialdehyde that converts spontaneously into pyrroline-5-carboxylate [25]. The latter is further reduced to proline by δ^1^-pyrroline-5-carboxylate reductase (P5CR). Both P5CS and P5CR use NADPH as reducing agent. Proline degradation occurs in mitochondria through the sequential action of two enzymes, proline dehydrogenase (PDH) and δ^1^-pyrroline-5-carboxylate dehydrogenase (P5CDH), regenerating reducing power in the form of enzyme-bound FADH_2_ and NADH, respectively. For this reason, both intracellular and intertissutal proline transport allows the transfer of reducing power [26].

As in most angiosperms, the *Arabidopsis* genome encodes two P5CS isoforms with only partially overlapping functions. The isoform 2 is mainly involved in proline production in absence of stress and its fundamental role is revealed by the embryo lethality of *p5cs2* mutant [27,28]. In contrast, *P5CS1* is responsible for the de novo accumulation of proline that occurs primarily in chloroplasts in response to stress or ABA treatments [27,29].

In the present study, the role of SUS1, GWD2 and P5CS1 has been analyzed in hydroponically grown *Arabidopsis* plants exposed to mild osmotic stress imposed through the addition of 150 mM mannitol. The aim was to study the fluctuations of the two metabolic pools of carbohydrates and amino acids, known to be strongly involved in the response of plants exposed to drought [30,31,32]. Several lines of evidence suggest an interaction between the two pools in response to osmotic stress.

## 2. Materials and Methods

### 2.1. Plant Material

Stock seeds of *sus1* line (At5g20830, SALK_014303C) were purchased from the European *Arabidopsis* Stock Center (NASC, Nottingham, U.K.). The homozygous T-DNA insertion was confirmed by two independent PCR amplifications on 400 ng of genomic DNA extracted from leaves of T3 plants grown on soil. The two pairs of primers were *sus1* fw 5′-CTCAAGAGTGCAAGGATCAGG-3′ plus *sus1* rev 5′-ACGCTGAACGTATGATAACGC-3′ and *sus1* rev 5′-ACGCTGAACGTATGATAACGC-3′ plus LBb1.3 5′-ATTTTGCCGATTTCGGAAC-3′, the first pair specific for the gene of interest and second pair specific for the gene of interest and the left border of T-DNA. The following condition was used for PCR amplifications in a Biometra T-gradient thermocycler: 5 min at 94 °C and 35 cycles of 30 s at 93 °C, 30 s at 58 °C and 1 min at 72 °C. PCR products were analyzed on a 0.8% agarose gel in 40 mM Tris-acetate buffer, pH 8.0, containing 10 mM EDTA and visualized with gel red (Figure S1). Homozygous T-DNA lines *gwd2* (At4g24450, SALK_080260C) and *p5cs1* (At2g39800, SALK_063517) have already been characterized in [24] and [33], respectively.

### 2.2. Plant Growth Conditions and Plant Harvests

If not differently specified, plants were hydroponically grown in a growth chamber not equipped with humidity control, at constant temperature of 22 °C, under a 12/12 h light/dark cycle with a photosynthetic photon flux density of approximately 110 μmoL m^−2^·s^−1^ measured at the level of the leaf surface, as described [34]. Osmotic stress was provided at 12 h dark by transferring 35-day-old plants into freshly prepared hydroponic medium (1.25 mM KNO_3_; 1.5 mM Ca(NO_3_)_2_ × 4H_2_O; 0.75 mM MgSO_4_ × 7 H_2_O; 0.5 mM KH_2_PO_4_; 50 μM H_3_BO_3_; 12 μM MnSO_4_ × H_2_O; 0.7 μM CuSO_4_; 1 μM ZnSO_4_ × 7 H_2_O; 0.24 μM MoO_4_Na_2_ × 2 H_2_O; 50 μM FeEDTA; 100 μM Na_2_SiO_3_ × 5 H_2_O) supplemented with 150 mM mannitol.

Rosettes were harvested at 12 h light under control and after 0.5 days after treatment (DAT), 4.5 DAT and 6.5 DAT. Plant materials were immediately ground with liquid nitrogen and the powders stored at −80 °C until use.

### 2.3. Determination of Water Content

For each time point, the water content was determined on 20–30 plants for each genotype harvested at 12 h light. The fresh weight (FW) of rosettes was recorded immediately after excision and the dry weight (DW) was recorded after 24 h of drying at 80 °C. Water content was calculated as the difference between the FW and the DW of each plant and expressed as a percentage of the FW.

### 2.4. Determination of Lipid Peroxidation

About 100 mg of frozen-ground material was used to measure lipid peroxidation by the 2-thiobarbituric acid (TBA) assay [35]. Three volumes of 0.1% (*w*/*v*) trichloroacetic acid (TCA) were mixed with frozen leaf powder. After centrifugation, 200 μL of supernatant was mixed with 800 μL of 20% (*w*/*v*) TCA and 5 μL of 0.5% (*w*/*v*) TBA. Samples were incubated for 30 min at 90 °C before the reaction was stopped by rapid cooling in ice water. Samples were spun, and the absorbance of supernatants was measured at 532 nm and 600 nm in a UV–Vis Cary60 (Agilent Technologies Inc., Santa Clara, CA, USA) spectrophotometer. The concentration of malondialdehyde (MDA) was calculated by subtracting non-specific absorption at 600 nm and using an extinction coefficient at 532 nm of 155 mM^−1^·cm^−1^. For each sample two technical repeats were measured. On average, five independent samples of 5–8 whole rosettes per sample were collected at each time point.

### 2.5. Leaf Starch Quantification

Leaf starch content was quantified as described in [36]. Briefly, in order to remove chlorophylls, 100–200 mg of frozen-ground material was washed in 80% (*v*/*v*) ethanol and boiled for 3 min. The ethanol wash was repeated until the samples turned white. Once the samples were bleached, the pellets were dried by evaporation and resuspended in 10 volumes of MilliQ^®^ water (Milli-Q Integral, Burlington, MA, USA). Suspension aliquots of 0.4–0.5 mL, depending on the weight of the powders, were transferred in clean 1.5 mL tubes and heated at 100 °C for 10 min. After cooling, an equal volume of 200 mM Na-acetate, pH 5.5, was added. Samples were mixed and split in two clean tubes. In one tube, 6 U of amyloglucosidase (Roche Diagnostics, Indianapolis, IN, USA) and 0.5 U of α-amylase (Roche) were added, while in the second tube, corresponding to the control sample, an equal volume of water was added. After overnight incubation at 37 °C, samples were centrifuged and the supernatants were assayed for glucose content by adding 1 U of hexokinase (Roche) and 1 U of glucose 6-phosphate dehydrogenase (Roche) in a reaction mixture containing 100 mM HEPES, pH 7.5; 0.5 mM ATP; 1 mM NAD^+^ and 4 mM MgCl_2_. For each sample a minimum of two technical repeats were measured at 340 nm in a UV–Vis Cary60 (Agilent Technologies) spectrophotometer. On average, five independent samples of 5–8 whole rosettes per sample were collected at each time point and analyzed.

### 2.6. Cell Wall Carbohydrates Quantification

Cell wall carbohydrates content was quantified as described in [37], with some modifications. Briefly, *Arabidopsis* frozen-ground material (about 100 mg) was washed 3 times with 1.5 mL 80% (*v*/*v*) ethanol and centrifuged to remove chlorophylls. Pellets were then washed 2 times with 1.5 mL 80% (*v*/*v*) acetone for 10 min and centrifuged again. To remove starch, dried pellets were resuspended in 0.5 mL of MilliQ^®^ water and gelled at 100 °C for 10 min. Samples were centrifuged and supernatants were discarded. Pellets were resuspended in 0.5 mL of 100 mM Na-acetate, pH 5.5, and the residual starch was degraded overnight by the addition of 6 U of amyloglucosidase (Roche) and 0.5 U of α-amylase (Roche) at 37 °C. Enzymes were inactivated at 100 °C for 10 min and samples spun down. Supernatants were discarded and pellets dried at 70 °C before adding 1 mL of Updegraff solution (glacial acetic acid:nitric acid (65% *v*/*v*):water solution in a 8:1:2 ratio; [38]). Resuspended samples were incubated at 100 °C for 30 min. After cooling, samples were spun down and supernatants discarded. Then pellets were washed once with 1.5 mL of MilliQ^®^ water, 3 times with acetone and dried overnight at room temperature.

Cell wall sugars were released by acid hydrolysis. The pellets were resuspended in 150 μL of 72% (*v*/*v*) H_2_SO_4_ and the samples were incubated for 45 min at room temperature, vortexing after 30 min of incubation. The acid hydrolysis was stopped with the addition of 600 μL of MilliQ^®^ water. Samples were centrifuged and the released sugars were quantified in the supernatants by Anthrone assay, mixing 100 μL of sample with 400 μL of Anthrone solution (1 mg·mL^−1^ Anthrone in 95% H_2_SO_4_). Samples were incubated at 100 °C for 5 min and then read at 630 nm using a plate reader (EnSpire Multimode Plate Reader, Perkin-Elmer, Beaconsfield, Buckinghamshire, UK). Glucose (0–50 μg) was used as a standard. On average, four independent samples of 5–8 whole rosettes per sample were collected at each time point and analyzed. Two technical repeats for each sample were measured.

### 2.7. Quantification of Total Water-Soluble Sugars

Total water-soluble sugars were extracted from *Arabidopsis* powders in a 1:2 ratio with cold MilliQ^®^ water. Extraction was performed three times by vortexing (1 min), centrifuging (15 min at 13,000 rpm) and collecting the supernatants in a single, clean tube. At the end of the extraction, total water-soluble sugars content was quantified by means of the Anthrone assay. Briefly, the supernatants were properly diluted in water and 350 μL of each sample was mixed with 700 μL of Anthrone solution. Samples were incubated 5 min at 100 °C and after cooling the absorbance at 630 nm was measured spectrophotometrically in 1 mL cuvettes (UV–Vis Cary60, Agilent Technologies). The standard curve was made with sucrose in a range of 0–50 μg. Two technical repeats for each sample were measured. On average, five independent samples of 5–8 whole rosettes per sample were collected at each time point and analyzed.

### 2.8. Glucose and Fructose Quantification

Glucose and fructose were extracted from 100 mg of frozen-ground material by three subsequent washes performed with an equal volume of cold MilliQ^®^ water. At each extraction, the samples were shaken vigorously for 1 min with a vortex, then centrifuged at 4 °C for 15 min at 13,000 rpm and the supernatants transferred to a clean tube. At the end of the extraction, the samples were incubated at 70 °C for 45 min and centrifuged for 10 min at 13,000 rpm in order to remove any debris. Glucose and fructose contents were evaluated using the Sucrose/D-Fructose/D-Glucose Assay Kit (Megazyme International Ireland, Co. Wicklow, Ireland) in a 96-well plate (EnSpire Multimode Plate Reader, Perkin-Elmer) following the manufacturer’s instructions. Two technical repeats for each sample were measured. On average, five independent samples of 5–8 whole rosettes per sample were collected every time point and analyzed

### 2.9. Proline and Amino Acids Quantification

Free amino acids and proline contents were quantified by the acid ninhydrin assay [39]. Leaf powders (about 100 mg) were mixed with 2 volumes of 3% (*w*/*v*) 5-sulfosalicylic acid and extracted by vortexing for 5 min at 1 min intervals. After extraction, the samples were centrifuged at 13,000 rpm for 10 min and the supernatants were transferred in clean tubes and spun again. Four technical repeats were assembled in a 96-well plate using from 5 to 15 μL of each sample. When necessary, the samples were brought to 15 µL with the addition of 3% 5-sulfosalicylic acid. After the addition of 15 μL of 3.0 M Na-acetate and 200 μL ninhydrin solution (0.15% (*w*/*v*) ninhydrin in glacial acetic acid), the background absorbance was read at 352 nm for proline and at 540 nm for total amino acid pool (T0) using a plate reader (EnSpire Multimode Plate Reader, Perkin-Elmer). The plate was then incubated at 50 °C for 12 min and allowed to cool for 3 min. Measures were repeated just after cooling (T15′) and after further 2.5 and 5 min (T17.5′ and T20′). T0 was subtracted from T15′, T17.5′ and T20′, and the average value was compared with standard curves to calculate proline and amino acids content. As standard, samples from 2.5 to 15 μL of a 25 mM mixture of amino acids solution similar to their abundance in plant extracts (5 mM glutamine, 2 mM glutamate, 2 mM aspartate, 1 mM the other proteinogenic amino acids, proline included—[40]) were measured. Analyses were performed on 3–4 independent samples of 5–8 whole rosettes per sample collected at each time point.

## 3. Results

### 3.1. Water Content under Osmotic Stress

**Figure 1:**
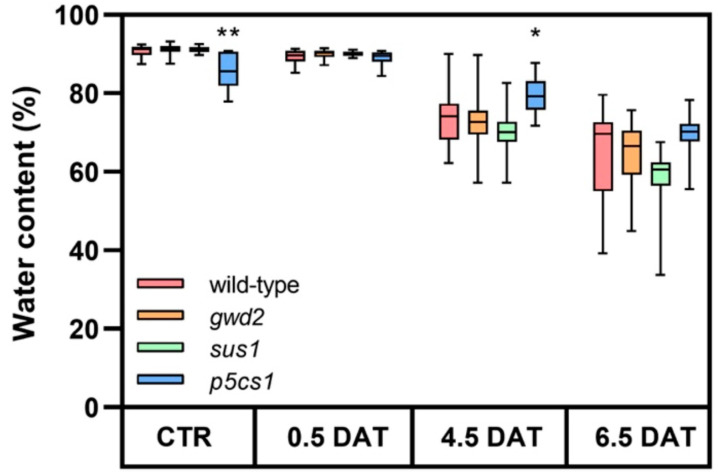
Relative water content (WC) under osmotic stress. Relative WC was calculated on whole rosettes cut from 20–30 plants for each genotype at each experimental point. Plants were collected at 12 h light under control condition (CTR) and at 0.5, 4.5 and 6.5 days after treatment (DAT). The fresh weights (FWs) were determined weighting plants immediately after the excision, while the dry weights (DWs) were determined after drying (24 h incubation at 80 °C). Water content was calculated as the difference between the FW and the DW of each plant and expressed as a percentage of the FW. Data are reported in boxplots, the central line represents the median and error bars the highest and the lowest data of the set. The *t*-test (wild-type vs. T-DNA lines) was used for statistics: * *p* < 0.01; ** *p* < 0.001. *p*-values are reported in Table S1.

To assess possible differences in osmotic compensation, the plant water content (WC) of each genotype under study was followed for 6.5 days by collecting samples at 12 h light over different days of treatment (Figure 1).

Mild osmotic stress was imposed to 35-day-old plants by adding 150 mM mannitol to the hydroponic medium. With the sole exception of *p5cs1*, which under control condition and at 4.5 DAT showed a slightly but significantly lower WC in comparison to wild-type plants (Table S1), after 6.5 days of treatment the WC in wild-type and all T-DNA plants was comparable, decreasing from 90% to about 65% of the FW (Figure 1).

Considering that all genotypes behaved similarly, in the subsequent experiments the metabolite content was normalized based on the FW.

### 3.2. Drought Leads to Different Oxidative Stress

**Figure 2:**
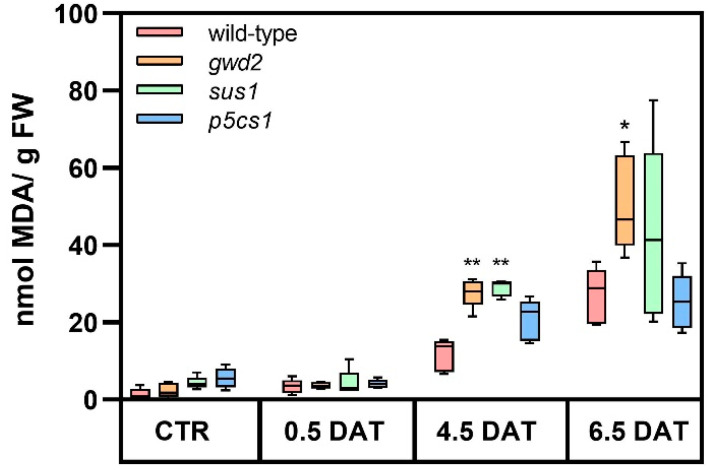
Lipid peroxidation under osmotic stress. The degree of stress induced by mannitol treatment was assessed by measuring lipid peroxidation in *Arabidopsis* rosettes collected at 12 h light under control condition (CTR) and at 0.5, 4.5 and 6.5 DAT. On average, 5 independent biological samples were analyzed for each experimental point. Data are reported in boxplots, the central line represents the median and error bars show the highest and the lowest data of the set. The *t*-test (wild-type vs. T-DNA lines) was used for statistics: * *p* < 0.01; ** *p* < 0.001. *p*-values are reported in Table S2.

Drought promotes the formation of reactive oxygen species (ROS) that interact directly with different macromolecules causing oxidative stress. In particular, the increase in ROS levels leads to lipid membranes oxidation with the subsequent formation of lipid hydroperoxides and their toxic aldehyde degradation products. The levels of lipid peroxidation, expressed as malondialdehyde (MDA) content, were measured in mannitol-treated plants. As shown in Figure 2, no difference was observed among the various genotypes under control conditions and at the onset of the osmotic stress (0.5 DAT). However, as the stress proceeded, the behavior of wild-type plants and T-DNA lines started to differ. At 4.5 DAT, the concentrations of MDA in *gwd2* and *sus1* plants were about double compared to wild-type plants. At 6.5 DAT, the difference was maintained only in *gwd2* plants, while the *sus1* line did not show significantly different levels with respect to wild-type plants (Table S2).

Unexpectedly, the degree of lipid peroxidation in the *p5cs1* line was similar to wild-type plants, probably due to the mild stress applied in the present study.

### 3.3. Effect of Drought on Carbohydrate Pools

**Figure 3:**
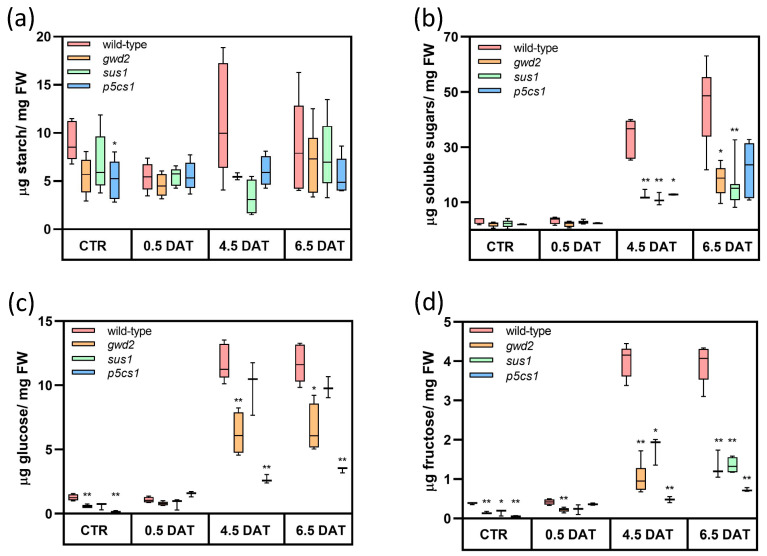
Carbohydrate pools rearrangements under water deprivation. Quantification of leaf starch (**a**), total water-soluble sugars (**b**), glucose pool (**c**) and fructose pool (**d**) in *Arabidopsis* plants grown under control condition (CTR) and exposed to mannitol treatment at 0.5, 4.5 and 6.5 DAT. Plants were collected at 12 h light. On average, 5 independent biological samples were analyzed for each experimental point. Data are reported in boxplots, the central line represents the median and error bars show the highest and the lowest data of the set. The *t*-test (wild-type vs. T-DNA lines) was used for statistics: * *p* < 0.01; ** *p* < 0.001. *p*-values are reported in Table S3.

In the metabolic effort to overcome the stress, primary starch mobilization is emerging as an important factor within plant responses [31,41]. Thus, primary starch accumulation was measured in *Arabidopsis* rosettes at the end of the day. As shown in Figure 3a, in all T-DNA lines the storage capacity of transitory starch at 12 h light was similar to wild-type plants and not significantly affected by the stress. On the contrary, the concentration of water-soluble sugars was strongly affected. In response to stress, levels in wild-type plants increased up to 15-fold at the end of the treatment. An increase was evident also in mutants, but much less pronounced (Figure 3b).

Focusing on the two main hexoses of the central metabolism, i.e., glucose and fructose, already under the control condition, all T-DNA lines had a lower concentration of both hexoses (Figure 3c,d). In this case also, a dramatic increase was evident in wild-type plants following the exposure to osmotic stress conditions, whereas the increase in the mutant lines was significantly lower (Table S3), except for glucose in *sus1* plants (Figure 3c). On the whole, the difference was higher for fructose than glucose. Interestingly, the most severe phenotype was observed in *p5cs1* mutant, the only mutant line not impaired in carbohydrate metabolism.

**Figure 4:**
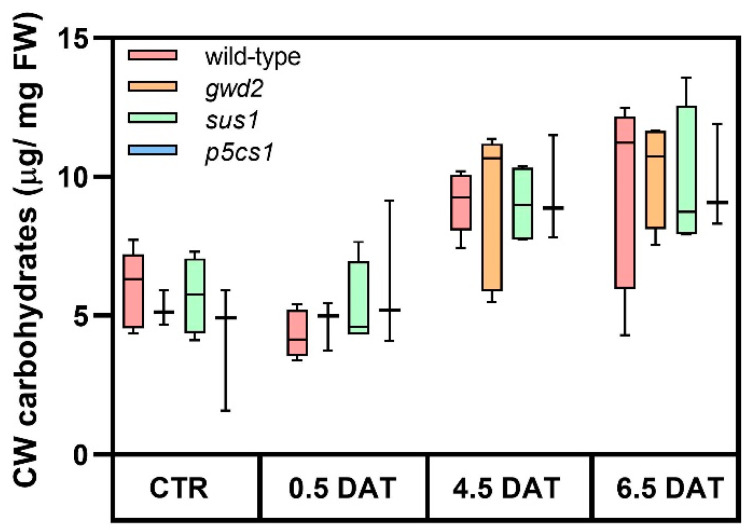
Cell wall (CW) carbohydrates. Quantification of insoluble non-starch carbohydrates from *Arabidopsis* plants grown under control condition (CTR) and exposed to mannitol treatment at 0.5, 4.5 and 6.5 DAT. *Arabidopsis* rosettes were collected at 12 h light. On average, 4 independent biological samples were analyzed for each genotype at each experimental point. Data are reported in boxplots, the central line represents the median and error bars show the highest and the lowest data of the set. The *t*-test (wild-type vs. T-DNA lines) was used for statistics. *p*-values are reported in Table S4.

Finally, considering that cell wall rearrangements have been reported to take part in adaptation to diverse abiotic and biotic stresses [42,43], the glucose deriving from the acid hydrolysis of the cell wall was quantified during the treatment (Figure 4).

Compared to wild-type plants, no differences were evident in the T-DNA lines, suggesting that GWD2, SUS1 and P5CS1 have no influence on cell wall metabolism, despite SUS1 can potentially produce UDP-glucose and therefore be involved in cellulose biosynthesis [44].

### 3.4. Effect of Drought on the Amino Acid Pools

**Figure 5:**
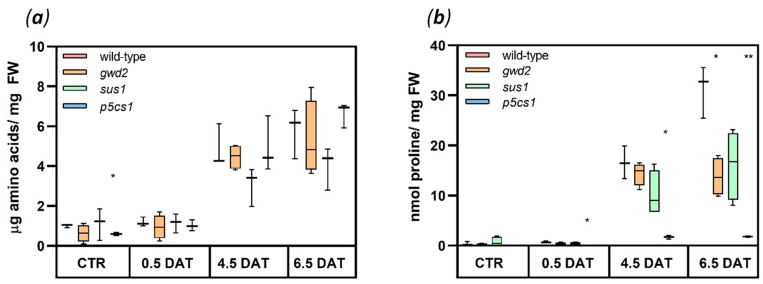
Amino acids and proline accumulation. Quantification of free amino acids (**a**) and proline (**b**) in *Arabidopsis* leaves collected at 12 h light under control condition (CTR) and at 0.5, 4.5 and 6.5 DAT. On average, 3 independent biological samples were analyzed for each genotype at each experimental point. Data are reported in boxplots, the central line represents the median and error bars show the highest and the lowest data of the data set. The *t*-test (wild-type vs. T-DNA lines) was used for statistics: * *p* < 0.01; ** *p* < 0.001. *p*-values are reported in Table S5.

The concentration of free amino acids was measured in control and stressed plants. The free amino acids levels in all lines increased along the stress without showing significant differences compared to wild-type plants (Figure 5a).

Among the proteinogenic amino acids, accumulation of proline is a well-documented response to several adverse conditions [27,45,46]. For this reason, proline accumulation can be assumed as an indication of proper response to stress. All genotypes, except *p5cs1*, accumulated proline in response to 150 mM mannitol treatment (Figure 5b). However, at 6.5 DAT, the increase was much less pronounced in *gwd2* and *sus1* lines, which accumulated only 44% and 52% of wild-type proline content, respectively (Figure 5b).

As expected, the concentration of free proline in the *p5cs1* mutant was extremely low already at the onset of the stress (0.5 DAT) (Figure 5b). These data are not surprising considering that the *P5CS1* gene is known to respond to water-limiting condition [27]. However, the data not only underline the role of P5CS1 in proline accumulation in response to drought but confirm that the homologous protein P5CS2 cannot replace P5CS1.

## 4. Discussion

Drought, high soil salinity and elevated temperatures are among the major causes of plant mortality affecting both crop yield and the maintenance of the natural heritage [47,48,49]. A recent hypothesis suggested that prolonged drought stress causes plant mortality through a combination of different factors, substantially attributable to carbon limitation and hydraulic failure, which together, rather than individually, lead to plant death [50]. Many different post-translational modifications eventually lead to cell death [51,52,53].

In consideration of the increase in the soluble sugars content observed in plants exposed to drought [54,55,56], the role played by the sole carbon starvation as the main metabolic determinant that triggers the transduction chains ending with the plant death appears less relevant. A possible explanation for such an increase is based on the fact that under moderate drought, plant growth declines before photosynthesis [55,56], resulting in an excess of carbon skeletons, which can be directed to osmolytes production [57,58,59].

In the present study, the analysis focused on metabolites acting as osmolytes or precursors for osmolyte production. Relying on the induction of the expression under drought or osmotic stress verified through eFP browser [60], three *Arabidopsis* single mutants, all members of gene families composed by two to six genes, were selected.

Under normal growth conditions, the pools of starch, total soluble sugars, cell wall carbohydrates and total amino acids were similar in all genotypes, although differences were specifically observed in glucose and fructose levels which were reduced in all mutants in respect to wild-type plants (Figure 3c,d). In response to stress, the non-soluble fraction of carbohydrates, here analyzed as transitory starch (Figure 3a) and cell wall carbohydrates (Figure 4), was unaffected by the mutations. The unchanged ability to accumulate leaf starch during the day is in agreement with the cytosolic localization of GWD2 and SUS1, and with the biological function of P5CS1, and suggests that none of the above enzymes causes a negative feedback loop, which acts on the ability of the chloroplast to accumulate starch. Similarly, the absence of GWD2, SUS1 and P5CS1 had no effect on the cell wall, although cell wall rearrangements have been reported to take part in the adaptation mechanisms that occur in both abiotic and biotic stress responses [42,43]. While this result was expected for *gwd2* and *p5cs1* mutants, the same was not true for *sus1* in view of the potential ability of SUS1 to produce UDP-glucose, which may be directed towards cellulose biosynthesis [44].

On the contrary, the pool of soluble sugars was largely affected by mannitol treatment. Soluble sugars are central components of energetic and biosynthetic metabolism, but they may act as well as both compatible osmolytes, re-establishing the osmotic balance, and protective macromolecules or scavengers of reactive oxygen species [61,62,63,64]. In comparison to wild-type plants, all mutants under study showed a lower capacity in soluble sugars accumulation (Figure 3b), with glucose and fructose as the most relevant components (Figure 3c,d). These findings further support the suggested role of SUS1 in the response to osmotic stress [15,16], and provide for the first time a role to GWD2 in stress response. However, an exhaustive comprehension of the phenotype shown by the *gwd2* mutant is complicated by the still unknown function of GWD2. It has been demonstrated that, in vitro, GWD2 phosphorylates glucans, likewise the plastidial isoform GWD1, which is fundamental for transitory starch degradation in chloroplasts [22,23]. However, in vivo evidence supporting a role for GWD2 in the mobilization of sugar from soluble and cytosolic substrates, such as phytoglycogen and soluble heteroglucans [65,66,67], remains scarce [24].

Considering the catalytic function of P5CS1 [45], the reduced capacity to accumulate soluble sugars could depend on the reduced ability of the *p5cs1* mutant to generate proline and therefore to regenerate NADP^+^ [26]. The reduction in NADP^+^ regeneration could overload the photosynthetic electron transport chain and compromise the production of soluble sugars.

Accumulation of both soluble sugars and proline takes part in the drought–response system [6,7,25,27] and it has been already suggested that the two signaling pathways interact [30,46]. This interaction has little influence on the total amino acids pool, rather it is specific for proline, whose strong increase under stress conditions is impaired in mutants (Figure 5a,b). However, we cannot exclude the simple explanation that the reduced capacity to accumulate proline observed in the *gwd2* and *sus1* mutants (Figure 5b) can be a mere consequence of the lower accumulation of glucose and fructose observed in these genotypes (Figure 3c,d). In fact, the lower concentration of hexoses could slow down the carbon flow through glycolysis diminishing the supply of pyruvate to tricarboxylic acids cycle with consequent reduction in glutamate production and thus proline synthesis. As a possible alternative, a reduced hexose availability could reduce NADPH production in the cytosol by the oxidative pentose phosphate pathway (which is early induced under osmotic stress [68]), affecting in turn proline production [69].

Considering that under stress conditions plants must produce osmolytes to protect the photosynthetic apparatus, maintain cell turgor and avoid a hydraulic failure, the results herein presented show that soluble sugars reach a concentration (about 260 nmol/mg FW) much higher than proline (concentration of 32 nmol/mg FW). In this respect, consistently with recent data on two halophytes [40], we can conclude that sugar metabolism gives a more substantial contribution to osmotic adjustment under drought stress. This notwithstanding, proline accumulation can benefit the cell through several other protective mechanisms [70].

## 5. Conclusions

Besides shedding light on the physiological role of GWD2 in response to drought, our results support a predominant role of soluble sugars in the stress response to mild osmotic stress in *Arabidopsis*. At least under the tested conditions, the accumulation of soluble sugars can be used to counteract osmotic stress more than proline. Although a putative interaction between proline and soluble sugars has emerged, the nature of this interaction remains to be investigated.

## Supplementary Materials

The following are available online at https://www.mdpi.com/2079-7737/9/11/367/s1, Figure S1: Selection of homozygous sus1 line by PCR analyses, Table S1: Statistics associated with Figure 1, Table S2: Statistics associated with Figure 2, Table S3: Statistics associated with Figure 3, Table S4: Statistics associated with Figure 4, Table S5: Statistics associated with Figure 5.
