# Supplementary material for: Impact of Drought on Soluble Sugars and Free Proline Content in Selected Arabidopsis Mutants
Source: Biology (Basel). 2020 Oct 29;9(11):367. doi: 10.3390/biology9110367 (PMC7692697; doi:10.3390/biology9110367)
Supplement: Supplementary file 1 [file biology-09-00367-s001.pdf]

biology

Article

Impact of Drought on Soluble Sugars and Free Proline Content in Selected *Arabidopsis* Mutants

Libero Gurrieri, Martina Merico, Paolo Trost, Giuseppe Forlani and Francesca Sparla

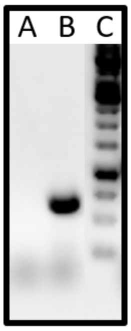

Figure S1. Selection of homozygous sus1 line by PCR analyses**.** PCR analyses were performed on genomic DNA. Specific primers and 35 amplification cycles were used for the selection. Representative result is reported. Lane A: PCR amplification performed with gene specific pair of primers (sus1 fw plus sus1 rev); lane B: PCR amplification performed with gene and T-DNA specific pair of primers (sus1 rev plus LBb1.3); lane C: DNA ladder (1 kb DNA ladder; Invitrogen).

Table S1. Statistics associated with Figure 1. *p*-value obtained from Student's *t*-tests performed on relative WC determined as described in Figure 1.

Treatment	gwd2	sus1	p5cs1
CTR	0.5075	0.2778	0.0000
0.5 DAT	0.1906	0.0857	0.3803
4.5 DAT	0.7909	0.0512	0.0021
6.5 DAT	0.9802	0.0295	0.0264

Table S2. Statistics associated with Figure 2. *p*-value obtained from Student's *t*-tests performed on degree of lipid peroxidation quantified as described in Figure 2.

Treatment	gwd2	sus1	p5cs1
CTR	0.3092	0.0171	0.0132
0.5 DAT	0.8820	0.6390	0.4534
4.5 DAT	0.0001	0.0001	0.0157
6.5 DAT	0.0015	0.1225	0.6560

Table S3. Statistics associated with Figure 3. *p*-value obtained from Student's *t*-tests performed on starch, soluble sugars, glucose and fructose concentration quantified as described in Figure 3.

Treatment	gwd2	sus1	p5cs1
	Starch		
CTR	0.0153	0.1852	0.0037
0.5 DAT	0.2585	0.8712	0.9742
4.5 DAT	0.1137	0.0195	0.0988
6.5 DAT	0.5432	0.6711	0.1473
	Soluble Sugars		
CTR	0.0391	0.1786	0.0182
0.5 DAT	0.1625	0.2744	0.2065
4.5 DAT	0.0005	0.0003	0.0026
6.5 DAT	0.0013	0.0001	0.0124

Glucose			
CTR	0.0002	0.0187	0.0006
0.5 DAT	0.0313	0.2758	0.0333
4.5 DAT	0.0009	0.1868	0.0000
6.5 DAT	0.0024	0.0925	0.0001
Fructose			
CTR	0.0000	0.0095	0.0000
0.5 DAT	0.0007	0.0467	0.5985
4.5 DAT	0.0000	0.0023	0.0000
6.5 DAT	0.0002	0.0000	0.0000

Table S4. Statistics associated with Figure 4. *p*-value obtained from Student's *t*-tests performed on CW carbohydrates concentration quantified as described in Figure 4.

Treatment	gwd2	sus1	p5cs1
CTR	0.4420	0.8112	0.2019
0.5 DAT	0.5459	0.2761	0.1884
4.5 DAT	0.9234	0.9247	0.7775
6.5 DAT	0.7410	0.9113	0.9078

Table S5. Statistics associated with Figure 5. *p*-value obtained from Student's *t*-tests performed on amino acids and free proline concentration quantified as described in Figure 5.

Treatment	gwd2	sus1	p5cs1
Amino acids			
CTR	0.1840	0.8356	0.0024
0.5 DAT	0.5534	0.9086	0.4847
4.5 DAT	0.5594	0.0989	0.9542
6.5 DAT	0.7236	0.1395	0.3607
Proline			
CTR	0.4557	0.6014	0.1585
0.5 DAT	0.0819	0.1343	0.0053
4.5 DAT	0.3415	0.0966	0.0014
6.5 DAT	0.0035	0.0258	0.0006

© 2020 by the authors. Licensee MDPI, Basel, Switzerland. This article is an open access article distributed under the terms and conditions of the Creative Commons Attribution (CC BY) license (http://creativecommons.org/licenses/by/4.0/).
